# Lactate metabolism–related differentially expressed genes reveal biological mechanisms underlying sarcopenia

**DOI:** 10.1038/s41598-025-25525-z

**Published:** 2025-11-24

**Authors:** Yan Li, Zhenbin Xu

**Affiliations:** 1 Department of Endocrinology, The Second Affiliated Hospital of Fujian Medical University, Quanzhou, 362000 Fujian Province China; 2 Department of Orthopaedics, The Second Affiliated Hospital of Fujian Medical University, Quanzhou, 362000 Fujian Province China

**Keywords:** Sarcopenia, Lactate metabolism, Differentially expressed genes, Gene ontology, Pathway enrichment, Biomarkers, Computational biology and bioinformatics, Diseases, Genetics

## Abstract

Sarcopenia (SARC), a progressive degenerative disorder associated with aging, is characterized by a gradual loss of muscle mass and strength and imposes major burdens on individuals and society. Current diagnostic and therapeutic methods for SARC remain limited, highlighting the urgent need for novel biomarkers. The present research was designed to investigate the role of lactate metabolism–related differentially expressed genes (LMRDEGs) in SARC, identify key genes and pathways, and develop a diagnostic model for the disease. Differentially expressed genes (DEGs) were identified using data from the Gene Expression Omnibus database. Gene set enrichment analysis was used to determine signaling pathways associated with the DEGs. Pearson’s correlation analysis was used to quantify the strength of linear relationships between coding genes and DEGs. Protein–protein interaction networks of DEGs were constructed using the STRING database. Functional annotation of DEGs was conducted using comprehensive enrichment analyses based on Gene Ontology (GO) terms and Kyoto Encyclopedia of Genes and Genomes (KEGG) pathways to elucidate their biological relevance. Seventeen LMRDEGs were found to be significantly upregulated or downregulated in SARC, underscoring their pivotal roles in disease pathogenesis. GO and KEGG pathway enrichment analyses revealed that these DEGs were primarily involved in metabolic energy regulation and intracellular signal transduction, suggesting their functional importance in SARC development. Immune infiltration analysis suggested substantial variations in immune cell abundance among SARC samples, emphasizing the immune system’s potential contribution to disease progression. This study demonstrates the importance of LMRDEGs in SARC and the need for further investigation into their roles as potential therapeutic targets.

**Supplementary Information:**

The online version contains supplementary material available at 10.1038/s41598-025-25525-z.

## Introduction

Sarcopenia (SARC), defined as age-related degenerative decline in muscle mass and functional capacity, presents a major public health challenge that primarily affects elderly individuals. It is associated with substantial deterioration in physical health and imposes a considerable economic burden on healthcare systems^1^. Current treatment options for SARC are limited, mainly involving physical exercise and nutritional interventions. Although these approaches offer benefits, their effectiveness can vary markedly among individuals, highlighting the urgent need for innovative biomarkers and diagnostic tools to facilitate early detection and targeted treatment of SARC.

SARC exhibits a multifaceted etiology, encompassing factors such as aging, chronic inflammation, and hormonal imbalances^2^. Moreover, emerging evidence has revolutionized our understanding of lactate’s contribution, shifting its perceived role from a metabolic byproduct to a key regulator of muscle function and disease processes^3^. Previous research has demonstrated that lactate metabolism is critical for muscle regulation, suggesting that lactate may act as a signaling molecule influencing muscle regeneration and adaptation^4^. Notably, disruptions in lactate metabolism have been associated with muscle atrophy and SARC^5,6^, prompting further investigation into lactate metabolism–related differentially expressed genes (LMRDEGs) and their role in SARC^7^. However, the heterogeneity of SARC makes it difficult to define its association with lactate metabolism^8^. Lactate not only functions as a byproduct of energy metabolism but also as a vital signaling molecule^9^. The expression patterns of its synthesis (*LDHA*), transport (*MCT1/4*), and utilization (*LDHB*) genes may vary across tissues^10^, complicating the identification of key targets in bioinformatics analyses. Moreover, current databases predominantly focus on inflammatory or apoptotic pathways in the omics data of patients with SARC, with limited coordinated annotation of lactate metabolism genes^11^. Constructing reliable coexpression networks from limited samples and validating the regulatory importance of lactate-related genes pose methodological challenges.

This study aimed to identify genes and molecular pathways contributing to SARC development and clarify the relationship between LMRDEGs and SARC. By developing a diagnostic model based on the identified biomarkers, we seek to improve early detection and therapeutic strategies for SARC.

## Materials and methods

### Data download

The SARC datasets GSE8479^12^ and GSE1428^13^ were downloaded from the Gene Expression Omnibus (GEO) database^14^ (https://www.ncbi.nlm.nih.gov/geo/) using the R package GEOquery^15^ (version 2.70.0). Samples of human (*Homo sapiens*) skeletal muscle tissue (vastus lateralis) were derived from both GSE8479 and GSE1428. The GSE8479 and GSE1428 datasets were generated usingGPL2700 and GPL96 microarray platforms, as shown in Table 1. The present study incorporated gene expression data from two independent datasets: GSE8479, including 25 SARC specimens and 26 matched controls, and GSE1428, containing 12 SARC cases and 10 control samples. All SARC and control groups were included in the analysis.

**Table 1:** GEO microarray chip Information.

	GSE8479	GSE1428
Platform	GPL2700	GPL96
Species	Homo sapiens	Homo sapiens
Tissue	Vastus lateralis	Vastus lateralis
Samples in SARC group	25	12
Samples in control group	26	10
Reference	PMID: 17,520,024	PMID: 15,687,482

GEO, Gene expression omnibus; SARC, Sarcopenia

 Lactic acid metabolism–related genes (LMRGs) were collected using the GeneCards database^16^ (https://www.genecards.org/), which offers extensive genomic annotations of human genes. Using “Lactate Metabolism” as the main search term and applying a minimum relevance score exceeding zero for protein-coding genes, we identified 56 LMRGs. In addition, a PubMed search (https://pubmed.ncbi.nlm.nih.gov/) using the same keyword led to the identification of 22 additional LMRGs from previously published studies^17^. Following data integration and elimination of redundant entries from the datasets, 73 unique LMRGs were identified (Table S1).

Batch effect correction across microarray datasets (GSE8479, GSE1428, and the merged GEO dataset) was conducted using the sva package (v3.50.0)^18^ in R. The final dataset comprised 37 SARC specimens and 36 matched controls. Preprocessing of the merged GEO dataset, including probe annotation, data standardization, and normalization, was performed using the limma package (v3.58.1)^19^. To evaluate the effectiveness of batch effect correction, principal component analysis (PCA) was applied to gene expression data before and after adjustment. PCA is a multivariate statistical method that reduces high-dimensional data into a lower-dimensional feature space using orthogonal eigenvectors, facilitating visualization of complex biological data in two- or three-dimensional coordinate systems while preserving maximal variance.

### Differentially expressed genes (DEGs) related to lactate metabolism in SARC

The study cohort from the integrated GEO dataset was systematically stratified into two groups: SARC cases and matched healthy controls. To identify DEGs across groups, we employed the limma package (version 3.58.1) in R. DEGs were defined as those with an absolute log2 fold change (logFC) of > 0 and a false discovery rate (FDR)-adjusted p value of < 0.05. Genes with positive logFC values (logFC > 0) and significant adjusted p-values (adj. *p* < 0.05) were classified as significantly upregulated. Conversely, genes with negative logFC values (logFC < 0) meeting the same significance threshold were categorized as downregulated. P value correction was conducted using the Benjamini–Hochberg (BH) method. Results were visualized as volcano plots generated using the ggplot2 package (version 3.4.4). To identify LMRDEGs associated with SARC, we conducted an intersection analysis between the DEGs from the combined GEO dataset (filtered by |log2FC| > 0 and adj. *p* < 0.05) and the previously identified LMRGs. The overlapping gene set was visualized using a Venn diagram. The visualization of gene expression patterns was performed using the pheatmap tool (version 1.2.2) implemented in R statistical software, and chromosomal localization was illustrated via the RCircos package (version 1.2.2)^20^.

To assess correlations among LMRDEGs, we applied the Spearman algorithm to analyze their expression patterns in the integrated GEO dataset. The correlation analysis results were displayed as a heatmap generated using the ggplot2 package (version 3.3.6). To further examine the differential expression of LMRDEGs between the SARC and control groups, we constructed a comparative expression plot based on their expression levels in the combined GEO dataset.

### Gene ontology (GO) and Kyoto encyclopedia of genes and genomes (KEGG) pathway analyses

GO^21^ analysis is a fundamental bioinformatics approach for functionally annotating gene sets by categorizing gene products into three domains: biological processes (BPs) characterizing molecular activities, cellular components (CCs) describing subcellular locations, and molecular functions (MFs) defining biochemical interactions. KEGG^22^ is a widely used bioinformatics resource that integrates data on genomic sequences, metabolic and signaling pathways, disease mechanisms, and pharmacological compounds. GO and KEGG enrichment analyses for LMRDEGs were performed using the clusterProfiler package (version 4.10.0)^23^ in R. These analyses were executed under stringent statistical criteria, including adj. *p* < 0.05 and FDR (q-value) < 0.05 as significance thresholds. Multiple testing correction was applied using the BH method.

### Gene set enrichment analysis (GSEA)

GSEA^24^ is a computational method used to determine whether predefined gene sets show statistically significant, coordinated differences between two biological states. In the present study, all genes from the merged GEO dataset were first ranked based on their logFC values by comparing SARC samples with control specimens. This ranked gene list was then analyzed via GSEA using the clusterProfiler package (version 4.10.0) in R, which enabled a systematic evaluation of pathway-level expression changes across the integrated datasets. Furthermore, this approach enabled a comprehensive assessment of coordinated gene expression patterns potentially contributing to observed phenotypic differences.

Next, SARC samples from the integrated GEO dataset were stratified into HighRisk and LowRisk groups based on the median least absolute shrinkage and selection operator (LASSO) RiskScore. Differential expression analysis was conducted utilizing the limma package (version 3.58.1) in R. Differential expression results were visualized through the utilization of the R package ggplot2 (version 3.3.6), and heatmaps were created with pheatmap package (version 1.0.12). For GSEA, genes from the SARC samples were again ranked by logFC values between the HighRisk and LowRisk groups. The ranked gene list was analyzed using clusterProfiler (version 4.10.0).

GSEA parameters included seed = 2022, minimum gene set size = 10, and maximum gene set size = 500. The Molecular Signatures Database (MSigDB) was used to access the c2 gene set (Cp. All.v2022.1.Hs). For statistical significance, we required an adj. p value of < 0.05 and an FDR of < 0.05. Multiple testing correction was implemented using the BH method to control type I errors.

### Gene set variation analysis (GSVA)

GSVA^25^ is a nonparametric, unsupervised method that converts sample-specific gene expression profiles into pathway-level enrichment scores, enabling the evaluation of pathway enrichment differences between groups. The h.all.v7.4.symbols.gmt gene set was acquired from MSigDB^26^. Subsequently, we conducted GSVA on the merged GEO dataset using the GSVA package (version 1.50.5) in R. This analytical approach enabled the quantification of differences in pathway activity between the SARC and control groups. We then applied the same gene signature on the integrated GEO dataset for functional enrichment analysis, comparing patients in the HighRisk and LowRisk groups. This comparison aimed to elucidate differential pathway activation patterns. Statistical significance was determined using a threshold of *p* < 0.05 for gene set enrichment.

### Construction of a diagnostic model for SARC

Logistic regression analysis was conducted to identify LMRDEGs and construct a diagnostic model for SARC based on the combined GEO dataset. When the dependent variable was binary (SARC vs. control), we analyzed the relationship between independent variables and the dependent variable using *p* < 0.05 as a significance threshold. A logistic regression framework was implemented to model these associations. Expression patterns of genes included in the regression model were visualized using forest plots.

Next, the support vector machine recursive feature elimination (SVM-RFE) algorithm^27^ was applied using the R package e1071 (v1.7.14) to screen potential biomarkers from the LMRDEGs identified via logistic regression. This algorithm iteratively removes the least informative features to improve classification performance.

Finally, we applied the LASSO method using the glmnet package (version 4.1.8) in R statistical software^28^, with parameters set to seed = 500) and family =“binomial” based on the LMRDEGs identified using the SVM-RFE algorithm. LASSO regression analysis, which applies a penalty term (λ × |β|), was employed to reduce model overfitting and improve generalizability. LASSO regression outcomes were graphically represented using diagnostic model plots and coefficient path plots. LASSO regression yielded a predictive model for diagnosing SARC, incorporating selected LMRDEGs. A LASSO risk score (RiskScore) was computed from the regression coefficients as follows:

$$\:\text{r}\text{i}\text{s}\text{k}Score\:=\:\sum\:_{i}Coefficient\:\left({gene}_{i}\right)\:\times\:\:mRNA\:Expression\:\left({gene}_{i}\right)$$

### Validation of the SARC diagnostic model

A nomogram^29^ is a visual tool that represents functional relationships among predictor variables using scaled line segments in a coordinate framework. According to the results of logistic regression analysis, a nomogram was developed using the rms package (version 6.7.1) in R to visualize associations among model genes. The predictive capability of the SARC diagnostic model was evaluated through a calibration plot constructed with LASSO regression, which enabled a quantitative evaluation of model accuracy and discriminative ability. To assess the clinical utility of the model, decision curve analysis (DCA) was performed using the ggDCA package^30^ (version 1.1) in R. This analysis provided a comprehensive evaluation of the model’s clinical utility in the integrated GEO dataset, focusing on the performance characteristics of the selected gene signatures.

Subsequently, receiver operating characteristic (ROC) curves were generated using the R package pROC^31^ (version 1.18.5), and area under the curve (AUC) values were calculated to assess the diagnostic performance of the LASSO-derived RiskScore. SARC samples were divided into HighRisk and LowRisk groups based on the median RiskScore from the diagnostic model. To explore gene expression differences between these groups, comparative expression profiles were generated. Finally, ROC curves were plotted, and AUC values were calculated for each individual model gene using the pROC package (version 1.18.5). AUC values range from 0.5 to 1.0, where 0.5–0.7 indicates low diagnostic accuracy, 0.7–0.9 suggests moderate accuracy, and > 0.9 reflects high accuracy. Thus, the closer the AUC is to 1, the better the model’s predictive performance.

### Protein–protein interaction (PPI) network

PPI networks represent complex systems of molecular interactions that regulate critical cellular processes, such as signal transduction, transcriptional regulation, metabolism, and cell cycle progression. Investigating these networks provides fundamental insights into protein function, disease-associated molecular pathways, and the intricate relationships between biomolecules within cellular systems. To establish a PPI network for the model genes, we utilized the STRING database^32^ (https://cn.string-db.org/) with a high-confidence interaction score threshold of 0.900. Highly interconnected modules within the PPI network were considered indicative of functionally relevant protein complexes. Genes showing significant interactions in the PPI network were identified as hub genes for further analysis.

To investigate the functional associations of hub genes, we used the GeneMANIA platform^33^ (https://genemania.org/), a bioinformatics resource applied to perform gene function predictions, gene list analysis, and candidate gene prioritization for subsequent experimental validation. The platform integrates multiple genomic and proteomic data sources to predict functionally related genes. This approach employs a weighted scoring system to prioritize datasets according to its relevance and infers gene functions through interaction patterns. The functional associations of hub genes were determined using GeneMANIA to construct an expanded PPI network incorporating genes with similar biological functions.

### Immune infiltration analysis of high and low risk groups

To characterize immune cell populations, we applied the single-sample GSEA (ssGSEA) method. This method included profiling multiple human immune cell subpopulations, such as activated CD8^+^ T cells, activated dendritic cells, γδ T cells, natural killer (NK) cells, and immunosuppressive regulatory T cells. Enrichment scores from ssGSEA were used to construct an immune cell infiltration profile that quantitatively characterized the distribution patterns of various immune cell populations in SARC samples obtained from the integrated GEO dataset. The ggplot2 package (version 3.4.4) in R was then used to visualize differences in immune cell expression between the LowRisk and HighRisk groups in the SARC samples. Significantly differentially expressed immune cells were identified for subsequent analyses.

Spearman’s rank correlation was used to evaluate relationships among immune cells, with results visualized via heatmaps constructed using pheatmap (version 1.0.12). In addition, correlations between hub genes and immune cells were analyzed using Spearman’s method and displayed as bubble plots generated via ggplot2 (version 3.4.4).

### Construction of a regulatory network

Transcription factors (TFs) play a pivotal role in modulating gene expression through interacting with hub genes in post-transcriptional processes. The ChIPBase database^34^ (http://rna.sysu.edu.cn/chipbase/) was used to identify TFs associated with hub genes. A TF–mRNA regulatory network was then constructed and visualized using Cytoscape^35^.

Micro RNAs (miRNAs) are crucial molecular regulators that govern fundamental BPs during development and evolutionary adaptation. They modulate multiple target genes, and individual genes may be targeted by several miRNAs. To explore potential regulatory interactions between hub genes and miRNAs, we used the StarBase v3.0 platform^36^ (https://starbase.sysu.edu.cn/) and subsequently constructed a gene–miRNA interaction network using Cytoscape.

RNA-binding proteins (RBPs)^37^ play essential roles in gene expression regulation, including transcriptional processing, differential splicing, post-transcriptional modifications, intracellular RNA trafficking, and protein synthesis. Using the StarBase v3.0 database^36^ (https://starbase.sysu.edu.cn/), we predicted RBPs targeting hub genes. Subsequently, Cytoscape software was utilized to construct and visualize the mRNA-RBP interaction network.

Finally, direct and indirect pharmacological targets associated with hub genes were identified using the Comparative Toxicogenomics Database (CTD)^38^ (https://ctdbase.org/). These drug–gene interactions were integrated into a comprehensive mRNA–drug regulatory framework, which was subsequently graphically represented through Cytoscape visualization software.

### Statistical analysis

All statistical analyses were conducted using R software (version 4.3.3). For continuous variables with normal distribution, independent two-sample *t*-tests were used. When analyzing non-normally distributed datasets, we utilized the Wilcoxon rank-sum test (also known as the Mann-Whitney U test) for two-group comparisons. For experimental designs involving multiple groups (three or more), the Kruskal-Wallis nonparametric test was applied. To evaluate potential relationships between molecular variables, we performed correlation analysis using Spearman’s rank correlation coefficient. Unless explicitly stated, all p-values were two-tailed, and statistical significance was defined as *p* < 0.05.

## Results

### Technology roadmap

**Fig. 1:**
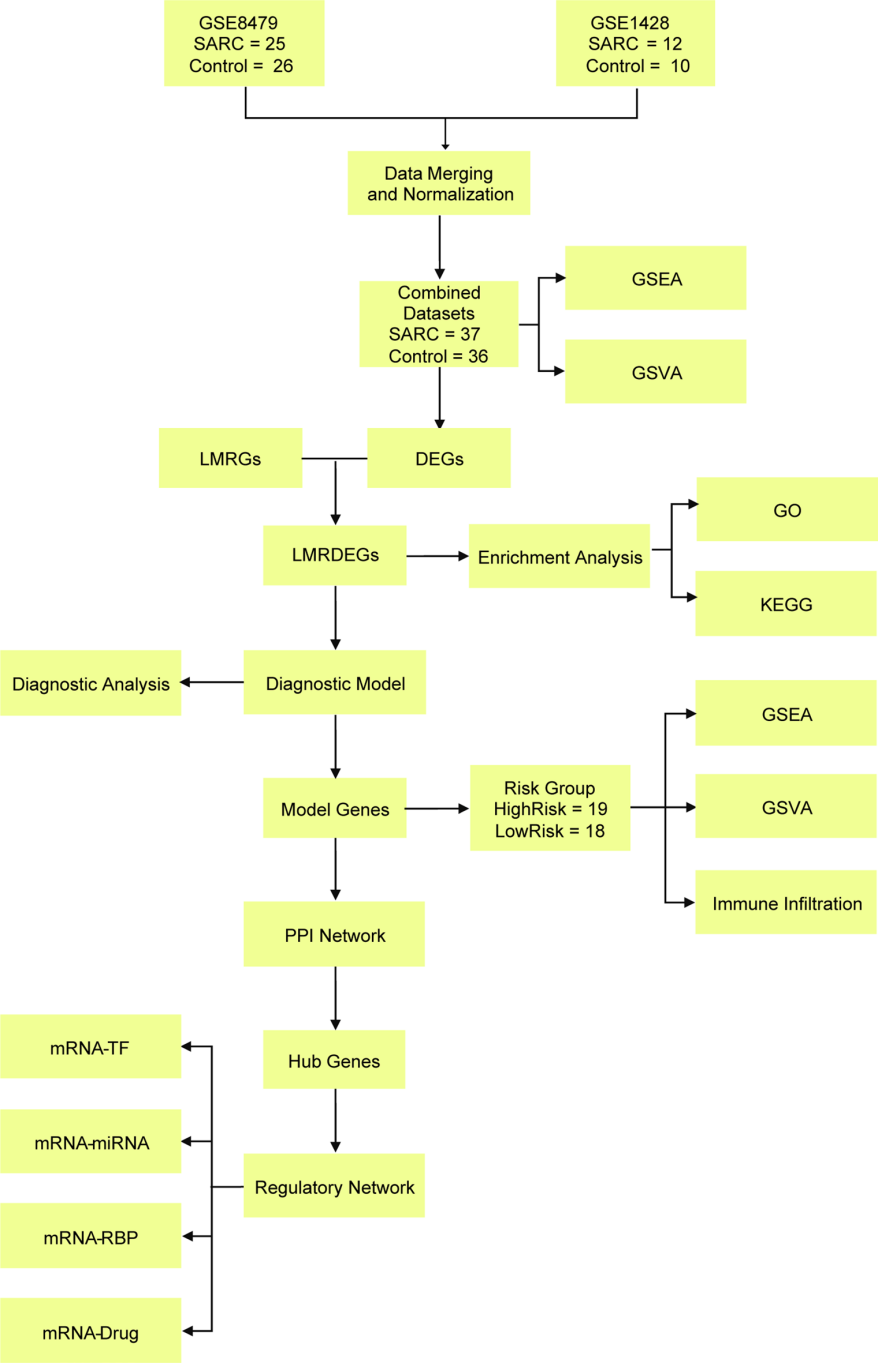
Flow chart for the comprehensive analysis of LMRDEGs. SARC, Sarcopenia; DEGs, Differentially Expressed Genes; LMRGs, Lactate Metabolism-Related Genes; LMRDEGs, Lactate Metabolism-Related Differentially Expressed Genes; GO, Gene Ontology; KEGG, Kyoto Encyclopedia of Genes and Genomes; GSEA, Gene Set Enrichment Analysis; GSVA, Gene Set Variation Analysis; PPI, Protein-Protein Interaction; TF, Transcription Factor; RBP, RNA-Binding Protein.

This study employed well-established bioinformatics tools and parameters (such as limma, sva, and ComBat) for data preprocessing, differential expression analysis, feature selection, and model construction (Fig. 1), ensuring that the results were scientifically valid and reproducible.

### Merging SARC datasets

**Fig. 2:**
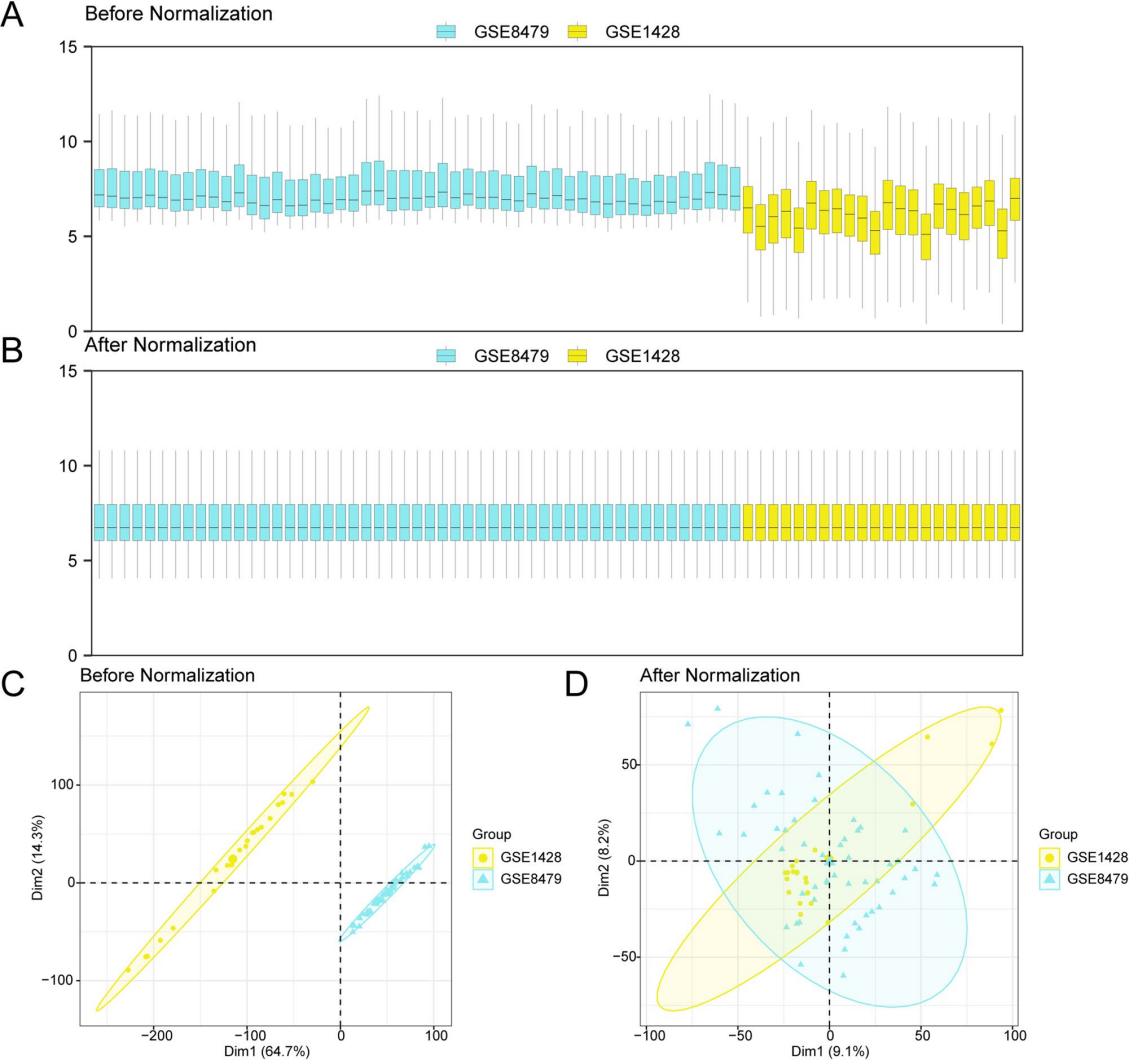
Batch Effects Removal of GSE8479 and GSE1428. (**A**) Box plot of Combined GEO Datasets distribution before batch processing. (**B**) Post-batch integrated GEO Datasets (Combined Datasets) distribution boxplots. (**C**) PCA plot of the datasets before debatching. (**D**) PCA plot of the Combined GEO Datasets following batch processing. PCA, Principal Component Analysis; SARC, Sarcopenia. The SARC dataset GSE8479 is represented in blue, while the SARC dataset GSE1428 is shown in yellow.

To construct an integrated dataset for SARC analysis, batch effects between the GSE8479 and GSE8479 datasets were removed using the sva package (version 3.50.0) in R, which yielded an integrated GEO dataset. Comparative analysis of expression profiles was performed through distribution boxplots (Fig. 2A, B), which illustrated the effectiveness of batch effect normalization. PCA was then employed to assess the spatial distribution of samples in reduced dimensions, with corresponding plots (Fig. 2C, D) illustrating the dataset structure before and after batch correction.

### DEGs related to lactate metabolism in SARC

**Fig. 3:**
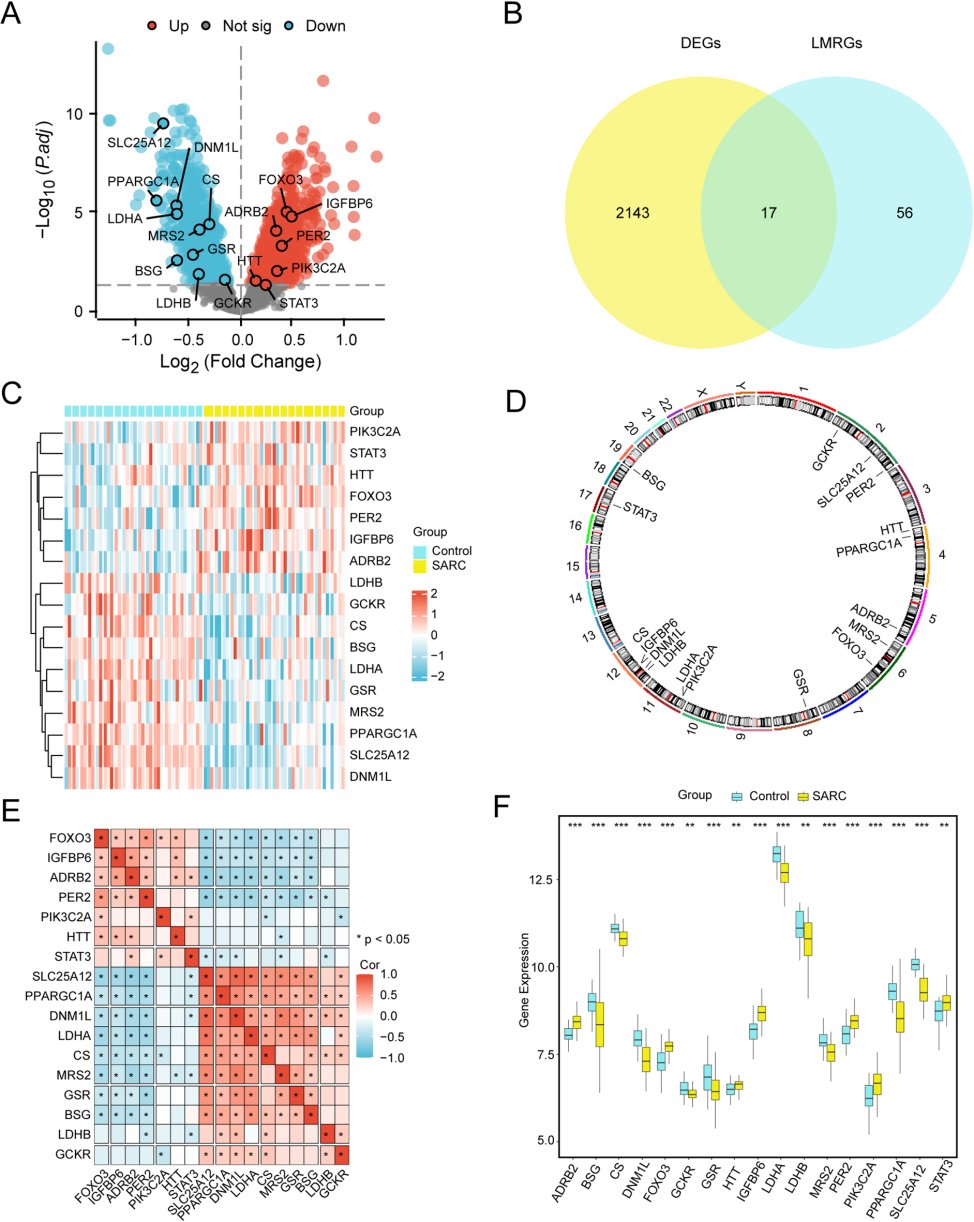
Differential Gene Expression Analysis. (**A**) Volcano plot of differentially expressed gene analysis between the SARC group and the Control group in the Combined GEO Datasets. (B) Venn diagram of DEGs and LMRGs in integrated GEO Datasets (Combined Datasets). (**C**) Heat map of LMRDEGs in the integrated GEO Datasets (Combined Datasets). (**D**) Chromosomal mapping of LMRDEGs. (**E**) Correlation heat map of LMRDEGs in Combined GEO Datasets. (**F**) Group comparison map of LMRDEGs in SARC and Control of integrated GEO Datasets (Combined Datasets). ** stands for p-value < 0.01, highly statistically significant; *** represents p-value < 0.001 and highly statistically significant. SARC, Sarcopenia; DEGs, Differentially Expressed Genes; LMRGs, Lactate Metabolism-Related Genes; LMRDEGs, Lactate Metabolism-Related Differentially Expressed Genes. In the heat map, the yellow color represents the SARC group, while blue denotes the Control group. Red indicates high expression levels, whereas blue signifies low expression levels. For the correlation heat map, red illustrates positive correlations, blue demonstrates negative correlations, and color intensity reflects the correlation strength.

The integrated GEO dataset was categorized into SARC and control cohorts, and comparative transcriptomic profiling was conducted using the limma package in R to identify DEGs. Based on the significance threshold (|logFC| > 0 and adj. *p* < 0.05), 2,160 significant DEGs were identified, including 1,119 upregulated genes and 1,041 downregulated genes. The differential expression landscape was visualized in a volcano plot (Fig. 3A). To detect LMRDEGs, we conducted a comprehensive intersection analysis between all significant DEGs and previously documented LMRGs. The results, shown in a Venn diagram (Fig. 3B), revealed 17 LMRDEGs: *FOXO3*, *IGFBP6*, *ADRB2*, *PER2*, *PIK3C2A*, *HTT*, *STAT3*, *SLC25A12*, *PPARGC1A*, *DNM1L*, *LDHA*, *CS*, *MRS2*, *GSR*, *BSG*, *LDHB*, and *GCKR.* The expression patterns of these LMRDEGs among different sample groups were analyzed in the integrated GEO dataset, with results presented in a heatmap generated via pheatmap (Fig. 3C). Chromosomal localization analysis, which was performed using RCircos (Fig. 3D), demonstrated that several LMRDEGs were clustered on chromosomes 12 (*CS*, *IGFBP6*, *DNM1L*, and *LDHB*) and 2 (*GCKR*, *SLC25A12*, and *PER2*).

Statistical analysis revealed significant intergenic correlations among the 17 LMRDEGs, as illustrated in a correlation heatmap (Fig. 3E). Most DEGs exhibited strong positive correlations, suggesting their potential functional relationships or coregulatory mechanisms. To further characterize the transcriptional profiles of LMRDEGs, we conducted a comparative analysis (Fig. 3F) to identify distinct expression patterns between SARC and control samples. In total, 13 LMRDEGs exhibited significant differential expression (*p* < 0.001): *FOXO3*, *IGFBP6*, *ADRB2*, *PER2*, *PIK3C2A*, *SLC25A12*, *PPARGC1A*, *DNM1L*, *LDHA*, *CS*, *MRS2*, *GSR*, and *BSG*. Furthermore, the LMRDEGs *HTT*, *GCKR*, *LDHB*, and *STAT3* showed significant differential expression (*p* < 0.01).

### GO and KEGG pathway enrichment analyses

**Fig. 4:**
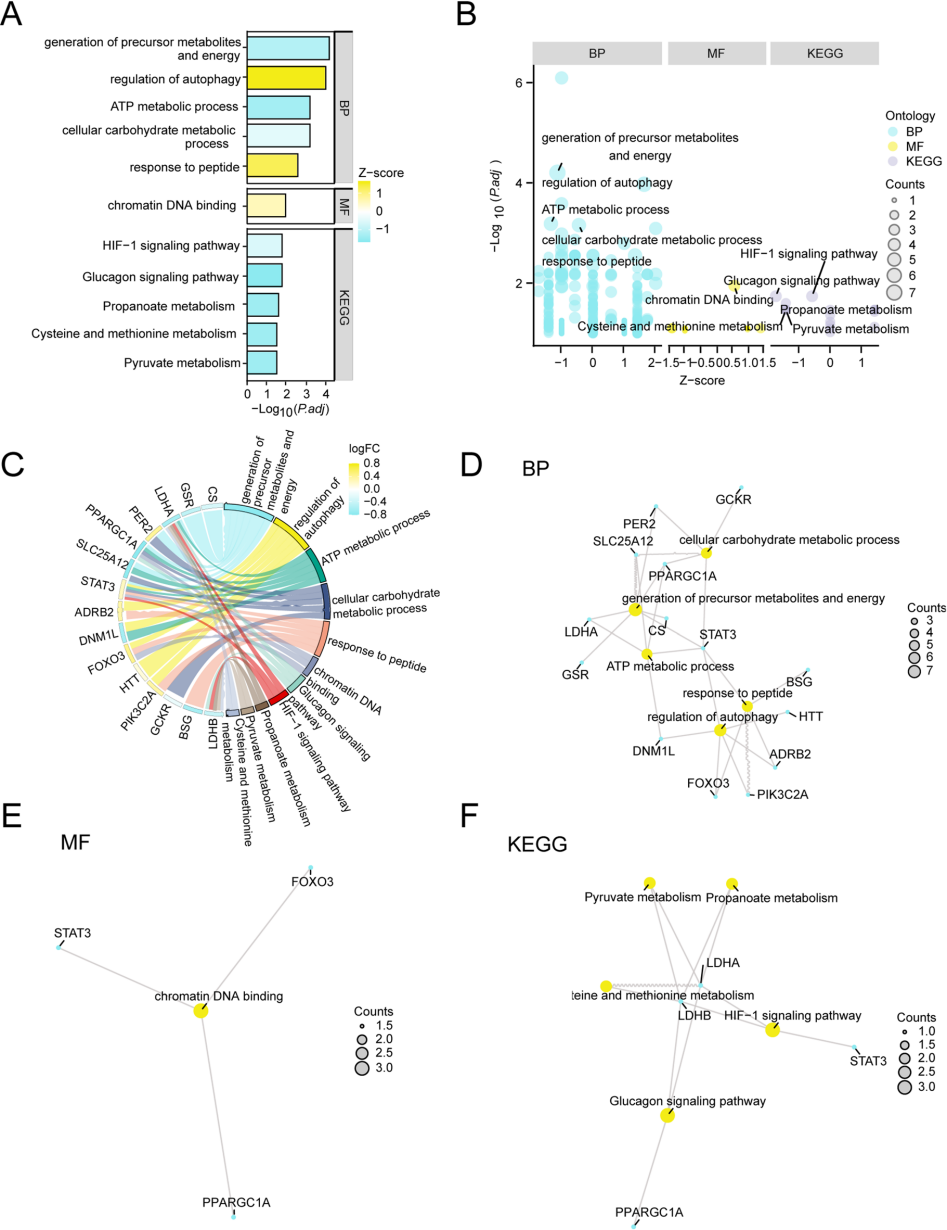
GO and KEGG Enrichment Analysis for LMRDEGs B. Bar graph of GO and KEGG enrichment analysis results of LMRDEGs: BP, MF and KEGG. B. Bubble plot of GO and KEGG enrichment analysis results of LMRDEGs. C. String diagram of GO and KEGG enrichment analysis results of LMRDEGs. D-F. GO and KEGG enrichment analysis results of LMRDEGs network diagram: BP (D), MF (E) and KEGG (F). Yellow nodes represent items, blue nodes represent molecules, and the connecting lines illustrate the relationships between items and molecules. LMRDEGs, Lactate Metabolism-Related Differentially Expressed Genes; GO, Gene Ontology; KEGG, Kyoto Encyclopedia of Genes and Genomes; BP, Biological Process; MF, Molecular Function. The screening criteria for GO and KEGG pathway enrichment analyses were an adjusted p-value < 0.05 and a FDR value (q-value) < 0.05, with the p-value correction method being BH.

To investigate the biological significance of the 17 LMRDEGs in SARC, we conducted GO and KEGG pathway enrichment analyses by integrating logFC values. These analyses enabled us to explore potential BPs, CCs, MFs, and associated signaling pathways. As demonstrated in Table 2, these LMRDEGs were enriched in BPs such as generation of precursor metabolites and energy, regulation of autophagy, and expression of genes involved in ATP metabolic process, cellular carbohydrate metabolic process, and response to peptide. For MFs, these genes were notably enriched in chromatin DNA binding. KEGG pathway analysis revealed enrichment in glucagon signaling pathway, HIF-1 signaling pathway, propanoate metabolism, pyruvate metabolism, and cysteine and methionine metabolism (Fig. 4A). The overall results, combined with logFC values, were further visualized using a bubble plot (Fig. 4B) and chord diagram (Fig. 4C). Network diagrams were also constructed to illustrate relationships among BPs, MFs, and KEGG pathways (Fig. 4D–F), where node size represented the number of associated molecules and connecting lines indicated specific molecular associations.

**Table 2:** Results of GO and KEGG enrichment analysis for LMRDEGs.

ONTOLOGY	ID	Description	Generatio	Bgratio	*p*-value	adj.*p*	q-value
BP	GO:0006091	generation of precursor metabolites and energy	7/17	494/18,800	1.28333E-07	6.20492E-05	3.40421E-05
BP	GO:0010506	regulation of autophagy	6/17	336/18,800	3.26719E-07	0.000105313	5.77778E-05
BP	GO:0046034	ATP metabolic process	5/17	273/18,800	3.33899E-06	0.000645761	0.000354285
BP	GO:0044262	cellular carbohydrate metabolic process	5/17	287/18,800	4.26318E-06	0.000687082	0.000376955
BP	GO:1,901,652	response to peptide	5/17	491/18,800	5.68035E-05	0.002762887	0.001515806
MF	GO:0031490	chromatin DNA binding	3/17	105/18,410	0.000115663	0.01110367	0.006452791
KEGG	hsa04922	Glucagon signaling pathway	3/12	107/8164	0.000441873	0.017963539	0.013015278
KEGG	hsa04066	HIF-1 signaling pathway	3/12	109/8164	0.000466585	0.017963539	0.013015278
KEGG	hsa00640	Propanoate metabolism	2/12	32/8164	0.000958646	0.024605247	0.017827452
KEGG	hsa00620	Pyruvate metabolism	2/12	47/8164	0.002063865	0.03346945	0.024249909
KEGG	hsa00270	Cysteine and methionine metabolism	2/12	51/8164	0.002426315	0.03346945	0.024249909

GO, Gene ontology; BP, Biological process; CC, Cellular component; MF, Molecular function; KEGG, Kyoto encyclopedia of genes and genomes; LMRDEGs, Lactate metabolism-related differentially expressed genes

### GSEA

**Fig. 5:**
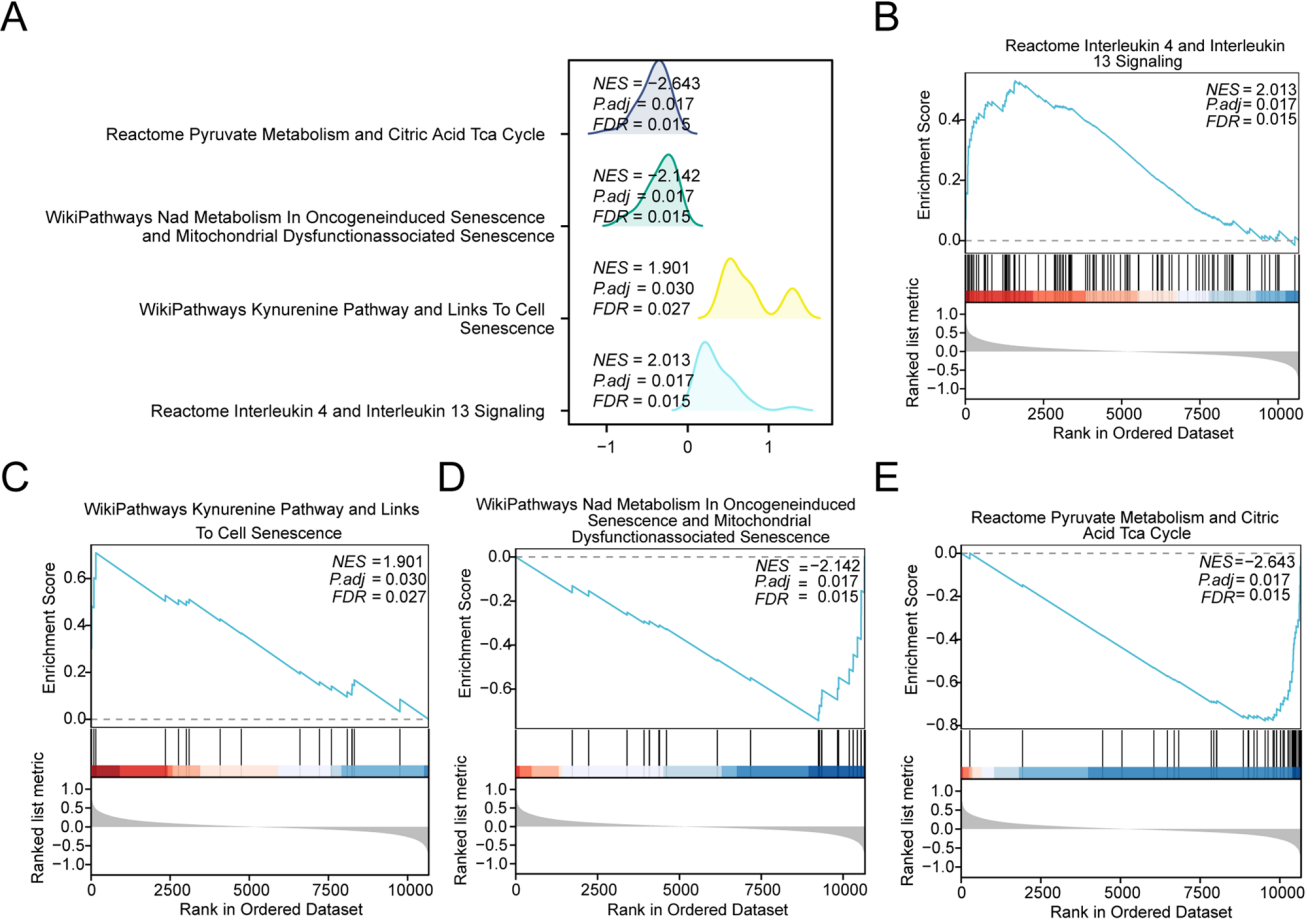
GSEA for combined datasets A. Mountain map presentation of 4 biological functions from GSEA of Combined GEO Datasets. B-E. GSEA showed that the Combined GEO Datasets were significantly enriched in INTERLEUKIN-4 AND INTERLEUKIN-13 SIGNALING (B), KYNURENINE PATHWAY AND LINKS TO CELL SENESCENCE (C), NAD METABOLISM IN ONCOGENEINDUCED SENESCENCE AND MITOCHONDRIAL DYSFUNCTIONASSOCIATED SENESCENCE (D), PYRUVATE METABOLISM AND CITRIC ACID TCA CYCLE (E). The screening criteria of GSEA were adj.p < 0.05 and FDR value (q-value) < 0.05, and the p-value correction method was BH.

To investigate the influence of transcriptional profiles obtained from the combined GEO dataset on SARC development, GSEA was performed using the logFC values of all genes, comparing SARC samples with controls. This approach allowed systematic exploration of associations between genome-wide expression signatures and corresponding biological pathways, cellular structures, and molecular mechanisms (Fig. 5A). Results are presented in Table 3.

**Table 3:** Results of GSEA for combined datasets.

ID	Set size	Enrichment score	NES	*p*-value	adj.*p*	q-value
INTERLEUKIN_4_AND_INTERLEUKIN_13_SIGNALING	102	0.530085438	2.013374534	0.000383877	0.017083693	0.015159772
KYNURENINE_PATHWAY_AND_LINKS_TO_CELL_SENESCENCE	18	0.709298332	1.90075194	0.001184366	0.030378064	0.026956965
COMPLEMENT_CASCADE	50	0.540009721	1.834070292	0.000763942	0.024393624	0.021646478
TYROBP_CAUSAL_NETWORK_IN_MICROGLIA	47	0.54834946	1.832609194	0.000762777	0.024393624	0.021646478
BMP2WNT4FOXO1_PATHWAY_IN_PRIMARY_ENDOMETRIAL_STROMAL_CELL_DIFFERENTIATION	12	0.755431385	1.83013082	0.001965409	0.038915094	0.034532578
FOXO_MEDIATED_TRANSCRIPTION_OF_CELL_DEATH_GENES	15	0.712423322	1.829252239	0.001564333	0.033485186	0.029714172
SMAD2_3NUCLEAR_PATHWAY	73	0.498472522	1.809851227	0.000754432	0.024393624	0.021646478
BIOCARTA_ETS_PATHWAY	14	0.716864789	1.808177328	0.002372479	0.045096085	0.040017481
NOTCH_PATHWAY	49	0.533667006	1.80222822	0.000762486	0.024393624	0.021646478
SA_G1_AND_S_PHASES	14	0.711158262	1.793783522	0.002372479	0.045096085	0.040017481
P53_TRANSCRIPTIONAL_GENE_NETWORK	77	0.487174073	1.785012916	0.000752445	0.024393624	0.021646478
HAIR_FOLLICLE_DEVELOPMENT_CYTODIFFERENTIATION_PART_3_OF_3	71	0.490986156	1.774873697	0.001126549	0.030378064	0.026956965
OVERLAP_BETWEEN_SIGNAL_TRANSDUCTION_PATHWAYS_CONTRIBUTING_TO_LMNA_LAMINOPATHIES	51	0.518803976	1.768155155	0.001526718	0.033485186	0.029714172
NAD_METABOLISM_IN_ONCOGENEINDUCED_SENESCENCE_AND_MITOCHONDRIAL_DYSFUNCTIONASSOCIATED_SENESCENCE	23	−0.74306012	−2.142357039	0.000412541	0.017083693	0.015159772
PYRUVATE_METABOLISM_AND_CITRIC_ACID_TCA_CYCLE	49	−0.777456953	−2.643079404	0.000420345	0.017083693	0.015159772

GSEA, Gene set enrichment analysis

GSEA revealed significant enrichment of genes from the integrated GEO dataset in several key pathways: interleukin-4 and interleukin-13 signaling (Fig. 5B), kynurenine pathway and links to cell senescence (Fig. 5C), NAD metabolism in oncogene-induced senescence and mitochondrial dysfunction-associated senescence (Fig. 5D), and pyruvate metabolism and citric acid TCA cycle (Fig. 5E), among other biologically relevant pathways and cellular functions.

### GSVA

**Fig. 6:**
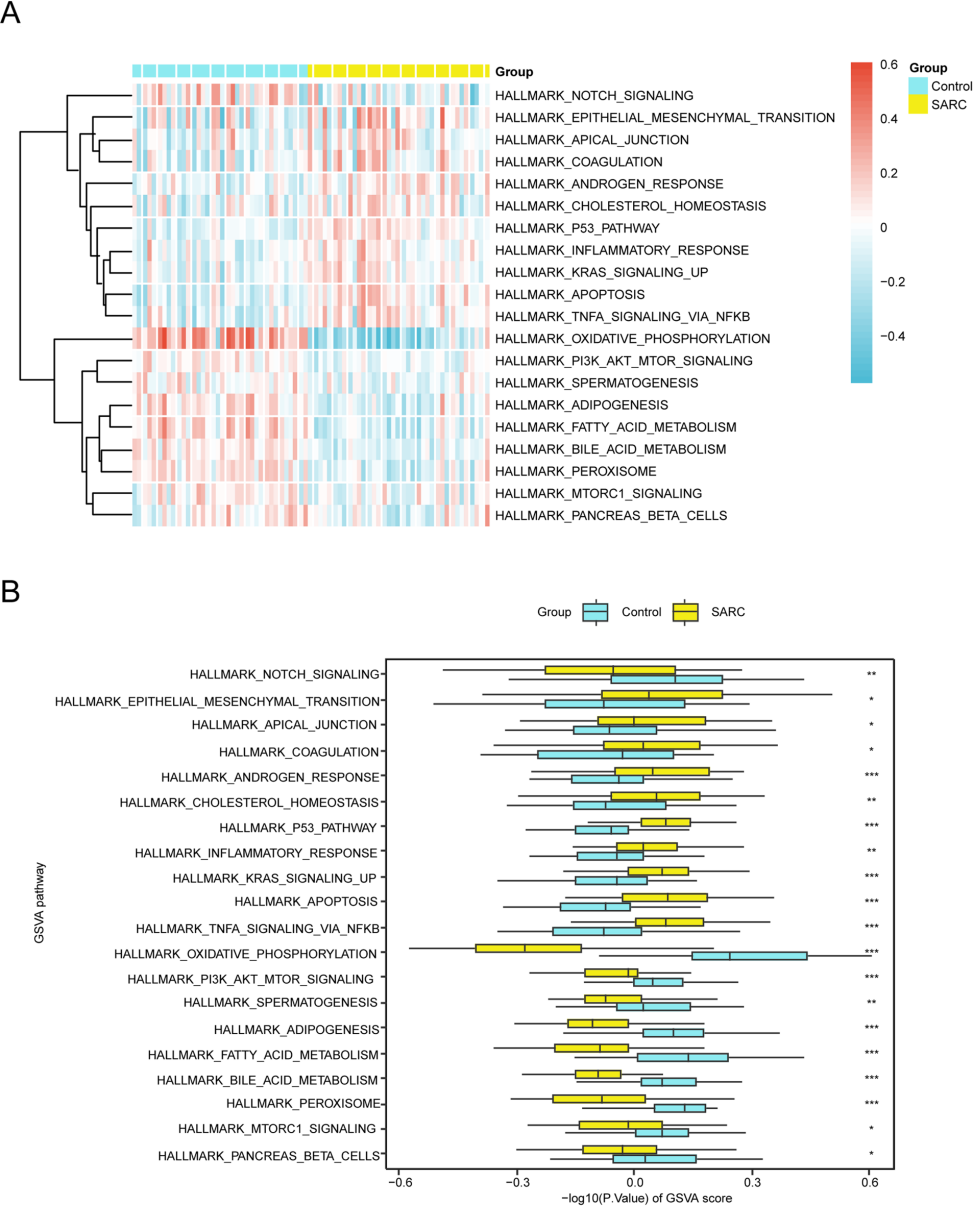
GSVA B-B. Heat map (A) and group comparison map (B) of GSVA results between SARC and Control groups of Combined GEO Datasets. SARC, Sarcopenia; GSVA, Gene Set Variation Analysis. * represents p-value < 0.05, statistically significant; ** represents p-value < 0.01, highly statistically significant; *** represents p-value < 0.001 and highly statistically significant. Yellow represents the SARC group and blue represents the Control group. The screening criterion for GSVA was a p-value < 0.05. Blue represents low enrichment and red represents high enrichment in the heat map.

To examine differences in the h.all.v7.4.symbols.gmt gene sets between the SARC and control groups, we conducted GSVA using the integrated GEO dataset (Table 4). Statistically significant pathways (*p* < 0.05) were systematically categorized according to their log2FC magnitude. The 20 most differentially expressed pathways (10 upregulated and 10 downregulated) were selected for further analysis. The differential expression patterns of these signaling cascades were analyzed and contrasted between the SARC and control groups, with the results graphically represented through a heatmap visualization (Fig. 6A). Statistical significance was confirmed through the Mann–Whitney U test, with the comparative analysis results visually presented in (Fig. 6B). GSVA identified multiple pathways with significant differential activity (*p* < 0.05) in SARC samples: OXIDATIVE PHOSPHORYLATION, FATTY ACID METABOLISM, ADIPOGENESIS, PEROXISOME, BILE ACID METABOLISM, NOTCH SIGNALING, PI3K AKT MTOR SIGNALING, MTORC1 SIGNALING, SPERMATOGENESIS, PANCREAS BETA CELLS, APICAL JUNCTION, INFLAMMATORY RESPONSE, CHOLESTEROL HOMEOSTASIS, KRAS SIGNALING UP, ANDROGEN RESPONSE, COAGULATION, EPITHELIAL MESENCHYMAL TRANSITION, P53 PATHWAY, TNFA SIGNALING VIA NFKB, APOPTOSIS.

**Table 4:** Results of GSVA for combined datasets.

ID	logFC	Aveexpr	t	*p*-value	adj.*p*	B
OXIDATIVE_PHOSPHORYLATION	0.532667	−0.000126	12.55144	0.000000	0.000000	36.77376
FATTY_ACID_METABOLISM	0.225002	0.006251	6.875144	0.000000	0.000000	11.31207
ADIPOGENESIS	0.188855	0.003316	6.236834	0.000000	0.000000	8.544764
PEROXISOME	0.173997	0.002921	5.791768	0.000000	0.000001	6.671987
BILE_ACID_METABOLISM	0.160146	−0.000200	6.676084	0.000000	0.000000	10.44030
NOTCH_SIGNALING	0.143962	0.013383	3.067677	0.002921	0.009737	−3.019175
PI3K_AKT_MTOR_SIGNALING	0.102818	0.001248	4.397415	0.000033	0.000181	1.245634
MTORC1_SIGNALING	0.084117	0.012006	2.632941	0.010111	0.024073	−4.156346
SPERMATOGENESIS	0.083731	−0.005312	3.006287	0.003507	0.010950	−3.188285
PANCREAS_BETA_CELLS	0.076525	−0.000974	2.301519	0.023895	0.049781	−4.924273
APICAL_JUNCTION	−0.084432	−0.001772	−2.270450	0.025798	0.051596	−4.991651
INFLAMMATORY_RESPONSE	−0.090485	−0.002681	−3.077619	0.002835	0.009737	−2.991531
CHOLESTEROL_HOMEOSTASIS	−0.101881	0.001687	−2.853857	0.005465	0.014380	−3.596243
KRAS_SIGNALING_UP	−0.113939	0.000589	−3.927990	0.000178	0.000888	−0.381275
ANDROGEN_RESPONSE	−0.115459	0.004598	−3.643640	0.000470	0.002134	−1.305700
COAGULATION	−0.119480	−0.003428	−2.889433	0.004934	0.013706	−3.502572
EPITHELIAL_MESENCHYMAL_TRANSITION	−0.121374	0.005798	−2.430391	0.017260	0.037522	−4.636254
P53_PATHWAY	−0.151852	−0.001811	−6.133916	0.000000	0.000000	8.107029
TNFA_SIGNALING_VIA_NFKB	−0.154492	0.000404	−4.611362	0.000015	0.000091	2.024977
APOPTOSIS	−0.180046	−0.002865	−5.787750	0.000000	0.000001	6.655333

GSVA, Gene set variation analysis

### Construction of a diagnostic model for SARC

**Fig. 7:**
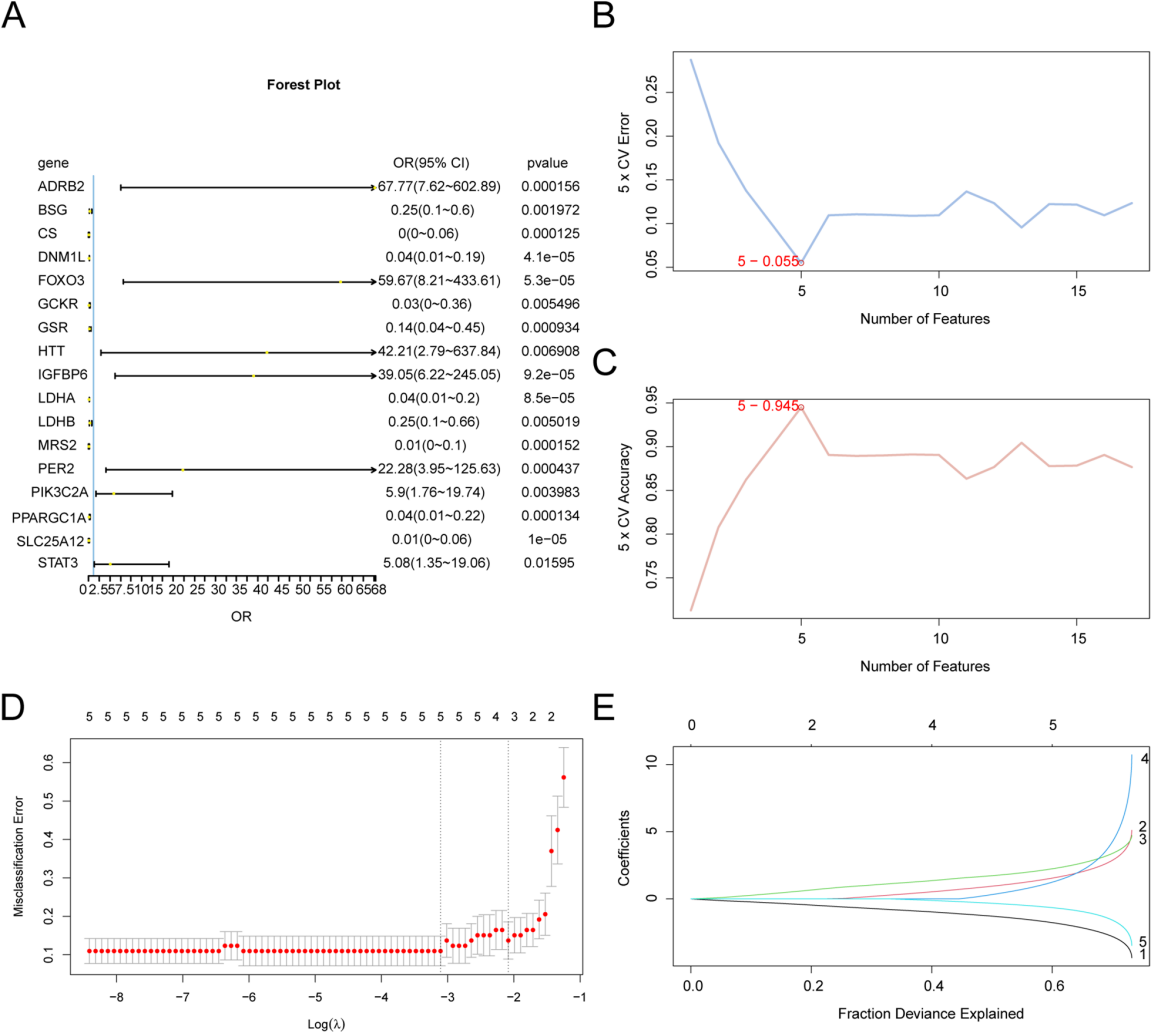
Diagnostic model of SARC**A**. Forest Plot of 17 LMRDEGs included in the Logistic regression model in the diagnostic model of sarcopenia. B-C. The number of genes with the lowest error rate (**B**) and the number of genes with the highest accuracy (C) obtained by SVM-RFE algorithm are visualized. D-E. Diagnostic model plot (**D**) and variable trajectory plot (**E**) of LASSO regression model. SARC, Sarcopenia; LMRDEGs, Lactate Metabolism-Related Differentially Expressed Genes; SVM, Support Vector Machine; LASSO, Least Absolute Shrinkage and Selection Operator.

To assess the diagnostic value of 17 LMRDEGs in SARC, we performed logistic regression analysis, with results visualized as a forest plot (Fig. 7A). All 17 LMRDEGs showed statistically significant associations (*p* < 0.05) within the regression model. Next, we applied the SVM-RFE algorithm with 3-fold cross-validation to the 17 LMRDEGs. Through iterative feature selection, we ranked genes and identified the optimal subset that minimized classification error (Fig. 7B) while maximizing predictive performance (Fig. 7C). The results indicated that the SVM model achieved peak accuracy when the following five genes, which consistently ranked highest, were incorporated: *PPARGC1A*, *PIK3C2A*, *FOXO3*, *HTT*, and *GSR*. We developed a LASSO regression–based diagnostic model for SARC incorporating these five LMRDEGs. Model performance was illustrated via regression coefficient plots (Fig. 7D) and variable selection trajectories (Fig. 7E). All five genes were confirmed as significant molecular markers contributing to the model’s diagnostic accuracy.

### Validation of the SARC diagnostic model

**Fig. 8:**
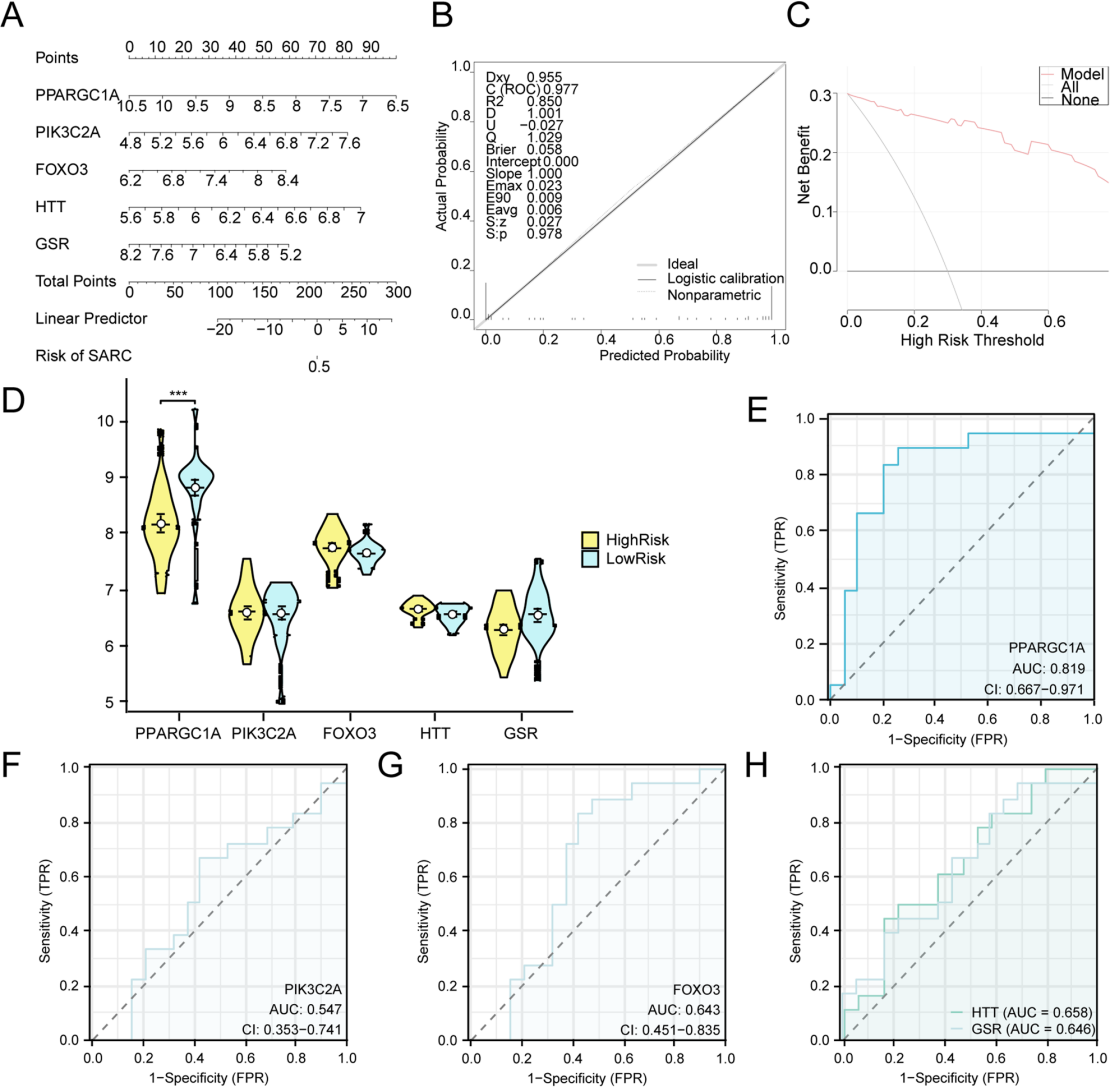
Diagnostic and validation analysis of SARC (**A**). Diagnostic nomograms of model genes in combined GEO datasets for SARC. B-C. Calibration curve (**B**) and decision curve analysis (**C**) of model genes in integrated GEO datasets (combined datasets) for SARC diagnosis. (**D**). Group comparison plots of Model Genes in HighRisk and LowRisk of SARC. E-H. ROC curves of model genes *PPARGC1A* (**E**), *PIK3C2A* (**F**), *FOXO3* (**G**), and *HTT/GSR* (**H**) in SARC. The DCA ordinate shows net benefit, while the abscissa represents threshold probability. SARC, Sarcopenia; DCA, Decision Curve Analysis; ROC, Receiver Operating Characteristic; AUC, Area Under the Curve; TPR, True Positive Rate; FPR, False Positive Rate. *** represents a p-value < 0.001 and highly statistically significant. When AUC > 0.5, it indicates that the expression of the molecule is a trend to promote the occurrence of the event, and the closer the AUC is to 1, the better the diagnostic effect. The AUC had some accuracy in the range of 0.7 to 0.9. Yellow represents HighRisk and blue represents LowRisk.

To assess the SARC model’s diagnostic value, we constructed a predictive nomogram incorporating the five model genes to visualize their interrelationships in the combined GEO dataset (Fig. 8A). Among the genes, *PPARGC1A* exerted the strongest influence, whereas *GSR* had the lowest impact on diagnostic scoring. The predictive accuracy and classification performance of the SARC model were assessed through calibration curve analysis, as illustrated in (Fig. 8B). Using the calibration curve, the concordance between observed event rates and model-predicted probabilities was analyzed across conditions. The calibration curve exhibited slight deviations from the perfect diagonal, yet maintained excellent overall agreement. DCA was used to quantify the clinical applicability of the SARC diagnostic model (Fig. 8C), demonstrating consistently higher net benefit across a range of thresholds compared with the “all-positive” and “all-negative” reference lines and thereby indicating superior clinical effectiveness.

Using the median RiskScore, patients were categorized into HighRisk and LowRisk groups. A statistical comparative of model gene expression between groups (Fig. 14D) revealed that *PPARGC1A* exhibited the most pronounced variation (*p* < 0.001). Finally, ROC curves were conducted using pROC to evaluate the diagnostic accuracy of each model gene (Fig. 14E–H). Results indicated moderate-to-high diagnostic performance for *PPARGC1A*, with AUC values between 0.7 and 0.9 across various comparisons.

### GSEA for highrisk and lowrisk groups

**Fig. 9:**
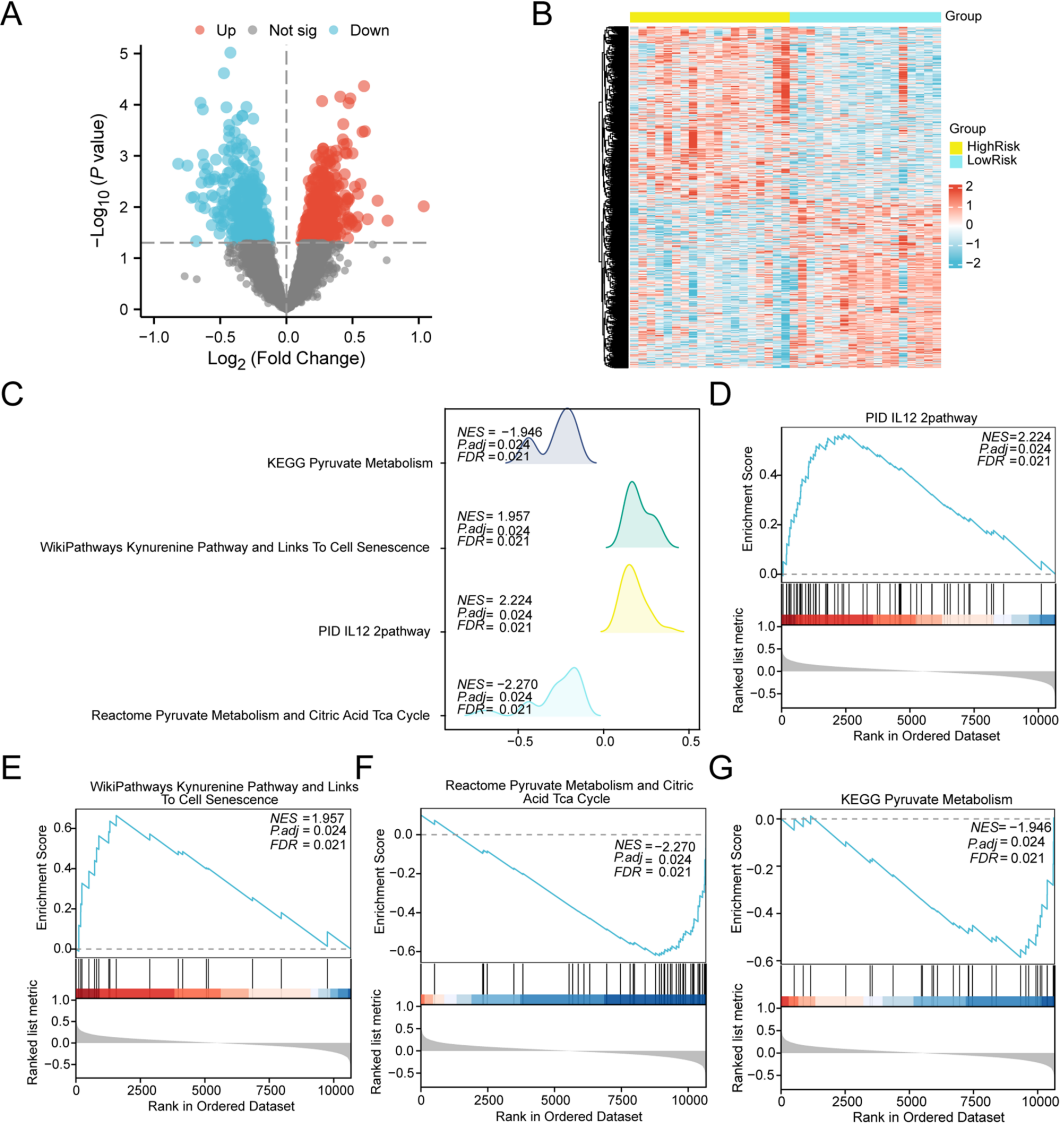
Differential Gene Expression Analysis and GSEA for SARC Sample B. Volcano plot (**A**) and heat map (**B**) of differentially expressed genes analysis in the HighRisk and LowRisk groups of SARC samples from the integrated GEO dataset. (**C**). GSEA of 4 biological function bubble plots of SARC samples. D-G. GSEA showed that the SARC samples from the integrated GEO dataset were significantly enriched in IL12 2pathway (**D**). Kynurenine Pathway and Links To Cell Senescence (**E**), Pyruvate Metabolism and Citric Acid Tca Cycle (**F**), Pyruvate Metabolism (**G**). SARC, Sarcopenia; GSEA, Gene Set Enrichment Analysis. Yellow represents the high-risk (HighRisk) group while blue represents the low-risk (LowRisk) group. In the heatmap, red indicates high expression and blue indicates low expression. The screening criteria for GSEA were adj.p < 0.05 and FDR, q-value < 0.05, with using the BH correction method.

To analyze differences among SARC samples, we stratified the integrated GEO dataset into HighRisk and LowRisk cohorts according to the median LASSO RiskScore. The limma-based differential expression analysis revealed 1,031 DEGs (absolute logFC > 0 with p-value < 0.05), comprising 521 genes showing increased expression and 510 downregulated genes. Results were visualized via a volcano plot (Fig. 9A) and pheatmap-generated heatmap (Fig. 9B).

To evaluate global gene expression impacts on SARC pathogenesis, we computed logFC values for all genes between risk groups. GSEA was conducted to investigate associations between gene expression patterns and BPs, with results visualized in a mountain plot (Fig. 9C) and summarized in Table 5. Significantly enriched pathways included IL12_2 pathway (Fig. 9D), kynurenine pathway (Fig. 9E), pyruvate metabolism and citric acid TCA cycle (Fig. 9F), and pyruvate metabolism (Fig. 9G).

**Table 5:** Results of GSEA for risk group.

ID	Set Size	Enrichment score	NES	*p*-value	adj.*p*	q-value
PYRUVATE_METABOLISM_AND_CITRIC_ACID_TCA_CYCLE	49	−0.621170043	−2.270049704	0.000367647	0.023645085	0.020961286
IL12_2PATHWAY	58	0.566247533	2.224086836	0.000442087	0.023645085	0.020961286
RACHIDONIC_ACID_METABOLISM	47	0.562425618	2.108191635	0.000437445	0.023645085	0.020961286
NONALCOHOLIC_FATTY_LIVER_DISEASE	129	−0.48440995	−2.101148722	0.000345423	0.023645085	0.020961286
OXIDATIVE_PHOSPHORYLATION	81	−0.489045571	−1.969498032	0.000358166	0.023645085	0.020961286
GLYCOLYSIS_AND_GLUCONEOGENESIS	44	−0.549955605	−1.960900456	0.00036914	0.023645085	0.020961286
KYNURENINE_PATHWAY_AND_LINKS_TO_CELL_SENESCENCE	18	0.663958378	1.957003611	0.000424268	0.023645085	0.020961286
PYRUVATE_METABOLISM	32	−0.585914054	−1.94601643	0.000372439	0.023645085	0.020961286
INTERLEUKIN_4_AND_INTERLEUKIN_13_SIGNALING	102	0.44468144	1.91839675	0.000467727	0.023645085	0.020961286
BIOCARTA_NO2IL12_PATHWAY	15	0.67762405	1.901194623	0.00041876	0.023645085	0.020961286
NEUTROPHIL_DEGRANULATION	384	0.37840427	1.946475313	0.000543183	0.026338835	0.023349287
METABOLIC_REPROGRAMMING_IN_COLON_CANCER	39	−0.538718599	−1.874240335	0.00073828	0.032774674	0.029054635
AMINO_ACID_METABOLISM	77	−0.432453124	−1.725839834	0.000723066	0.032774674	0.029054635
VITAMIN_B12_METABOLISM	40	0.523782582	1.894773095	0.00087146	0.034152407	0.03027599
ARACHIDONIC_ACID_METABOLISM	45	0.506506142	1.877756036	0.00087604	0.034152407	0.03027599

GSEA, Gene set enrichment analysis

### GSVA in highrisk and lowrisk groups

**Fig. 10:**
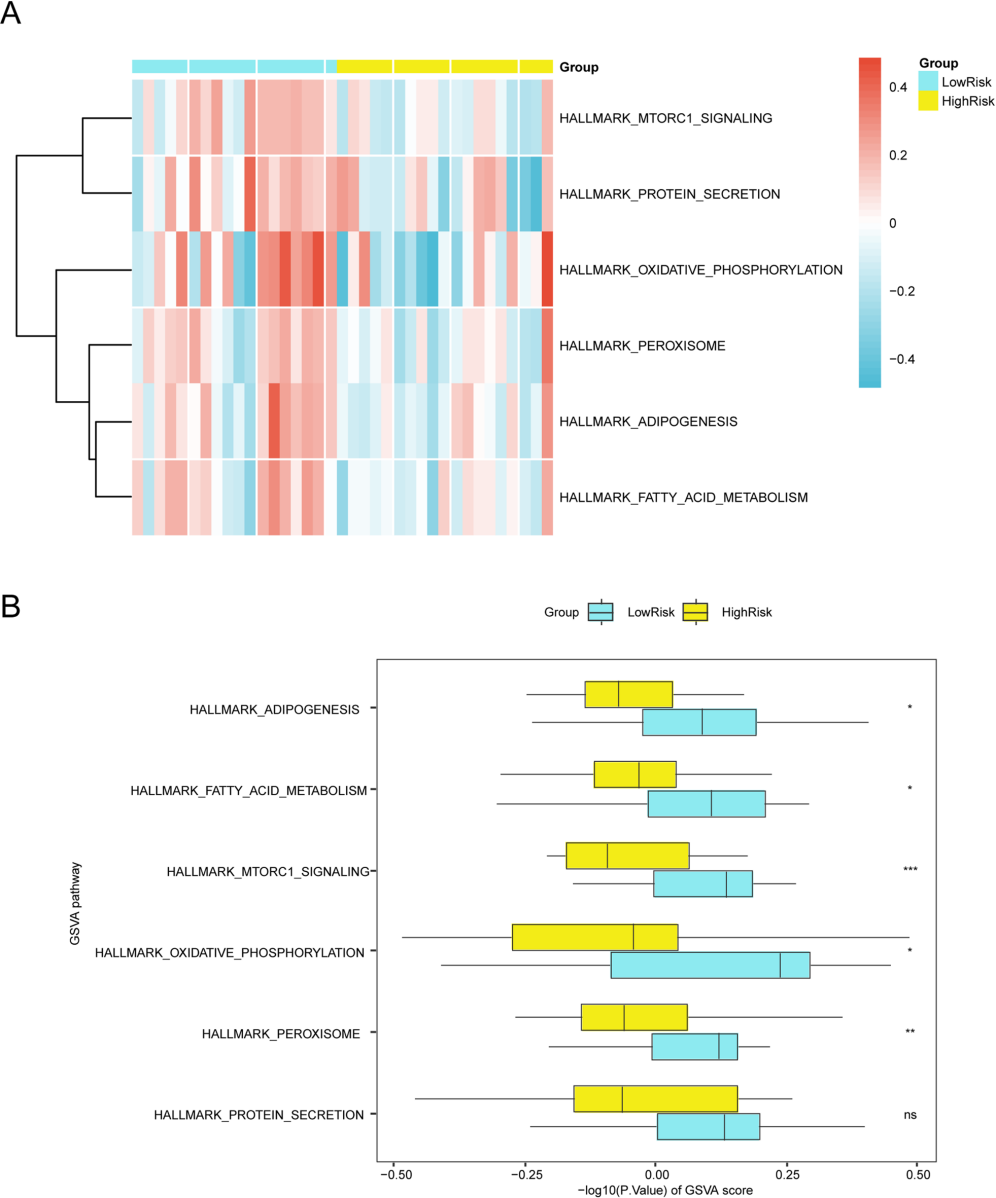
GSVA for SARC sample A-B. Heat map (**A**) and group comparison map (**B**) of GSVA results between HighRisk and LowRisk groups of SARC samples from the integrated GEO dataset. SARC, Sarcopenia; GSVA, Gene Set Variation Analysis. ns stands for p-value ≥ 0.05, not statistically significant; * represents p-value < 0.05, statistically significant; ** represents p-value < 0.01, highly statistically significant; *** represents p-value < 0.001 and highly statistically significant. Yellow indicates the HighRisk group, while purple denotes the LowRisk group. The screening threshold for GSVA was set at P < 0.05. In the heatmap, blue signifies low enrichment and red represents high enrichment.

To explore pathway-level differences between the HighRisk and LowRisk SARC groups, GSVA was performed on all genes using the h.all.v7.4.symbols.gmt gene set (Table 6). Six significantly altered pathways (*p* < 0.05) were identified based on descending absolute logFC values (Fig. 10A). The results were further validated using the Mann–Whitney U test and visualized in a comparative group plot (Fig. 10B). The following pathways exhibited statistically significant differences between the HighRisk and LowRisk groups: ADIPOGENESIS, FATTY_ACID_METABOLISM, MTORC1_SIGNALING, OXIDATIVE_PHOSPHORYLATION, and PEROXISOME.

**Table 6:** Results of GSVA for risk group.

ID	logFC	Aveexpr	t	*p*-value	adj.*p*	B
PEROXISOME	−0.112077	0.005078	−2.374395	0.021950	0.253951	−3.395191
ADIPOGENESIS	−0.117102	0.017172	−2.313087	0.025395	0.253951	−3.515193
FATTY_ACID_METABOLISM	−0.120356	0.009235	−2.463940	0.017671	0.253951	−3.215681
PROTEIN_SECRETION	−0.142890	0.028487	−2.156539	0.036479	0.303995	−3.810525
MTORC1_SIGNALING	−0.145698	0.016058	−3.226169	0.002354	0.117692	−1.505522
OXIDATIVE_PHOSPHORYLATION	−0.191631	0.015229	−2.403147	0.020484	0.253951	−3.338097
PEROXISOME1	−0.112077	0.005078	−2.374395	0.021950	0.253951	−3.395191
ADIPOGENESIS1	−0.117102	0.017172	−2.313087	0.025395	0.253951	−3.515193
FATTY_ACID_METABOLISM1	−0.120356	0.009235	−2.463940	0.017671	0.253951	−3.215681
PROTEIN_SECRETION1	−0.142890	0.028487	−2.156539	0.036479	0.303995	−3.810525
MTORC1_SIGNALING1	−0.145698	0.016058	−3.226169	0.002354	0.117692	−1.505522
OXIDATIVE_PHOSPHORYLATION1	−0.191631	0.015229	−2.403147	0.020484	0.253951	−3.338097

GSVA, Gene set variation analysis

### PPI interaction network

**Fig. 11:**
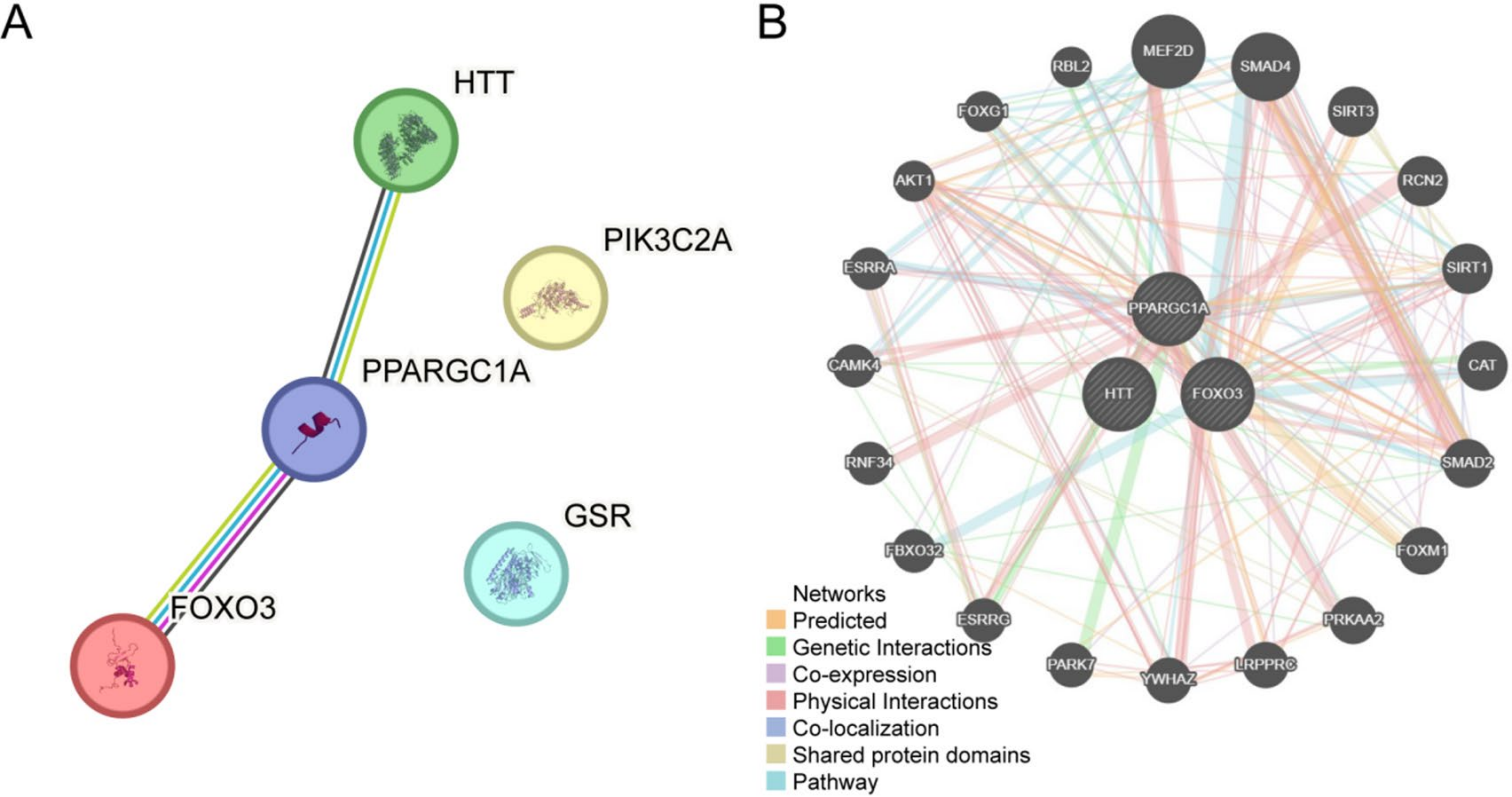
PPI network analysis**A**. PPI of model genes generated by STRING database. **B**. Functional interaction network of hub genes and their functionally similar counterparts predicted by GeneMANIA. In the figure, circles represent hub genes and their functionally related counterparts, while the colored connecting lines indicate different interaction types. PPI, protein-protein interaction network.

To investigate PPIs involving the five candidate genes, we established a PPI network using the STRING database (Fig. 11A), which revealed that *PPARGC1A*, *FOXO3*, and *HTT* were interconnected as hub genes. Further analysis using GeneMANIA extended the network to include these hub genes and 20 functionally associated proteins (Fig. 11B). Interaction types, including coexpression and shared protein domains, have been depicted using color-coded edges.

### Immune infiltration analysis (ssGSEA) of highrisk and lowrisk groups

**Fig. 12:**
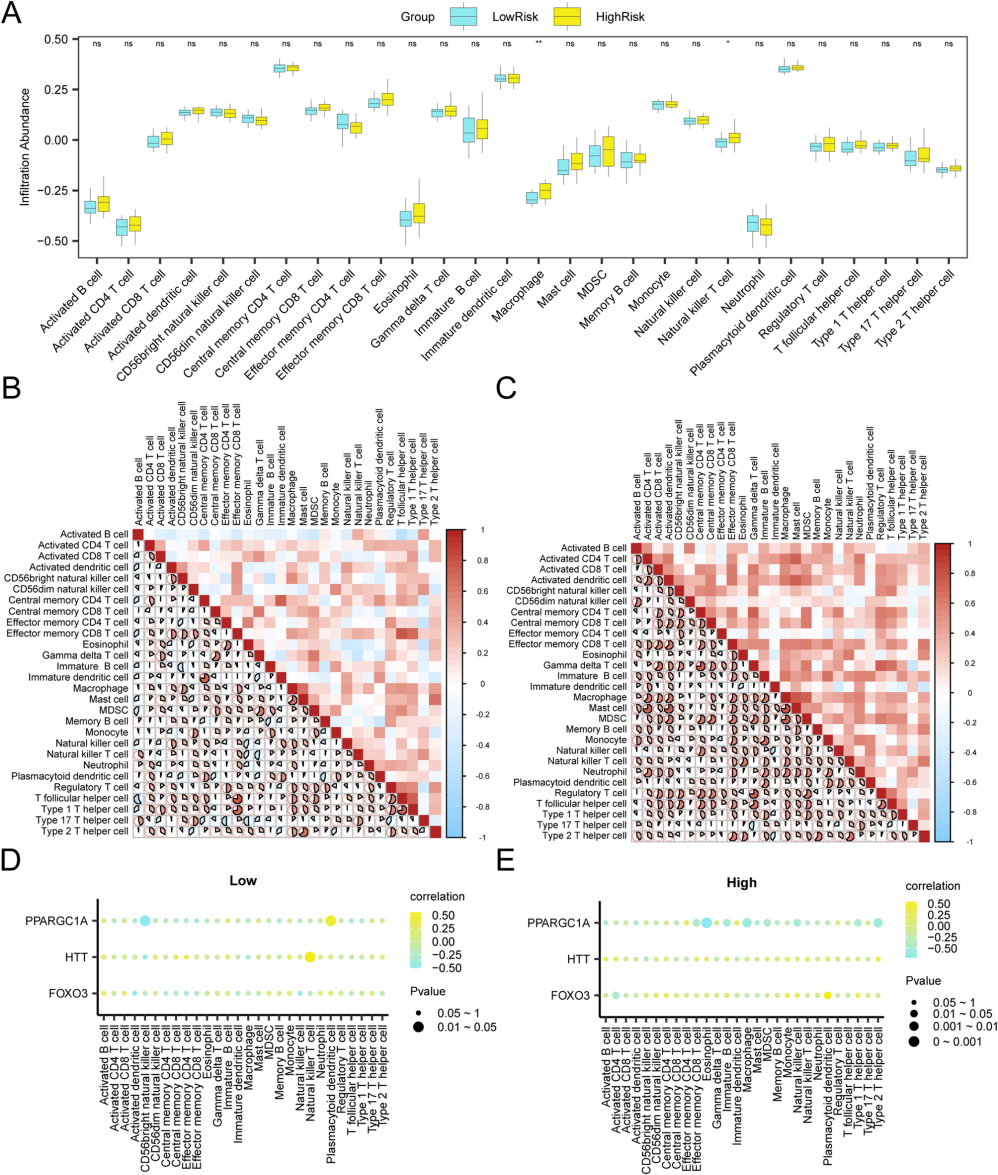
Risk group immune infiltration analysis by ssGSEA algorithm A. comparison of the grouping of immune cells in the lowrisk group and highrisk group of SARC samples. **B**-**C**. Results of correlation analysis of immune cell infiltration abundance in the LowRisk (**B**) and HighRisk (**C**) groups of SARC samples are shown. **D**-**E**. Bubble plot of correlation between immune cell infiltration abundance and hub genes in the LowRisk (**D**) and HighRisk (**E**) groups of SARC samples. ssGSEA, single-sample Gene-Set Enrichment Analysis; SARC, Sarcopenia. ns stands for p-value ≥ 0.05, not statistically significant; * represents p-value < 0.05, statistically significant; ** represents p-value < 0.01 and highly statistically significant. The absolute value of correlation coefficient (r value) below 0.3 was weak or no correlation, between 0.3 and 0.5 was weak correlation, and between 0.5 and 0.8 was moderate correlation. Blue is the LowRisk group, and yellow is the HighRisk group. Yellow is a positive correlation, blue is a negative correlation, and the depth of the color represents the strength of the correlation.

To evaluate the infiltration characteristics of immune cells in SARC samples obtained from the combined GEO dataset, we employed ssGSEA to systematically measure the proportional representation of 28 immune cell types. Comparative analysis examining intergroup variations in immune infiltration levels (Fig. 12A) revealed significant differences in immune infiltration (*p* < 0.05), particularly among macrophages and NK cells. Correlation heatmaps illustrated interrelationships among all immune cell types (Fig. 12B, C). We also investigated correlations between hub gene expression and immune cell infiltration, with findings shown in bubble plots (Fig. 12D, E). Notably, *HTT* was positively correlated with NK cells in the LowRisk group (*r* = 0.55, *p* < 0.05; Fig. 12D), whereas *FOXO3* showed a positive correlation with plasmacytoid dendritic cells in the HighRisk group (*r* = 0.54, *p* < 0.05; Fig. 12E).

### Construction of the regulatory network

**Fig. 13:**
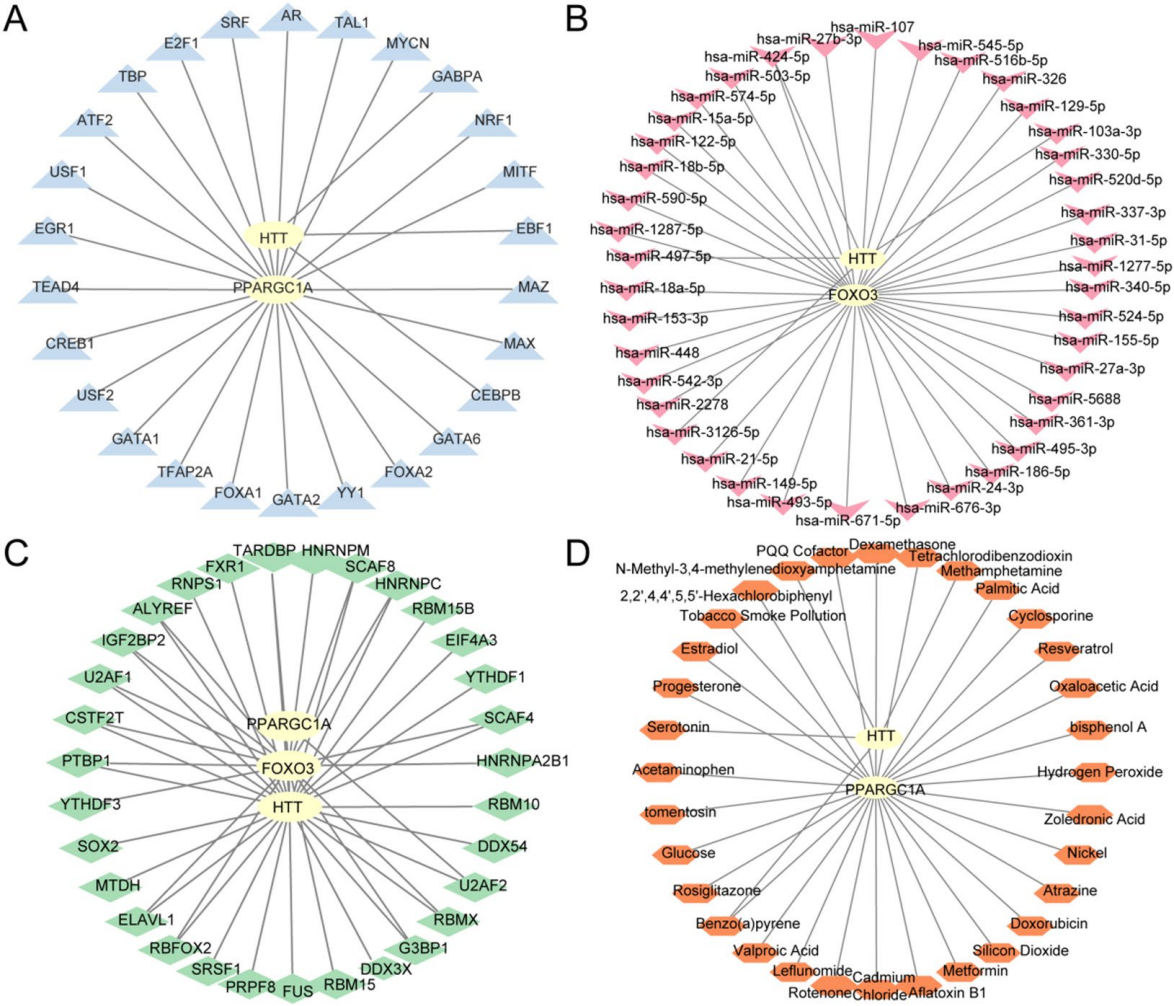
Regulatory network of Hub genes **A.** mRNA-TF Regulatory Network of hub genes. **B**. mRNA-miRNA Regulatory Network of hub genes. **C**. mRNA-RBP Regulatory Network of hub genes.**D**. mRNA-Drug Regulatory Network of hub genes. TF, Transcription Factor; RBP, RNA-Binding Protein. Yellow is mRNA, blue is TF, pink is miRNA, green is RBP, and orange is Drug.

 To elucidate the regulatory mechanisms of hub genes, we analyzed their interactions with various biomolecules. Initially, TFs potentially binding to hub genes were predicted through the ChIPBase database, after which an mRNA–TF regulatory network was constructed using Cytoscape (Fig. 13A), with this network incorporating 2 hub genes and 26 TFs (Table S2). Subsequent analysis focused on microRNA-mediated regulation, identifying miRNAs potentially targeting hub genes via the StarBase database. The resulting mRNA–miRNA interaction network (Fig. 13B) revealed 2 hub genes potentially regulated by 41 distinct miRNAs (Table S3). For post-transcriptional regulation analysis, RBPs interacting with hub genes were also predicted using StarBase. The mRNA–RBP regulatory network (Fig. 13C) revealed interactions between 3 hub genes and 31 RBPs, with molecular details provided in Table S4. Finally, we queried the CTD to identify 32 drug compounds targeting 2 hub genes, forming an mRNA–drug interaction network (Fig. 13D) comprising the hub genes and pharmacologically active compounds (Table S5).

## Discussion

SARC is a degenerative condition characterized by the progressive loss of muscle mass and function. This age-associated pathology has emerged as a major public health concern, particularly affecting the elderly. Despite its growing public health impact, early diagnostic tools remain lacking^39^, underscoring the need for reliable biomarkers and therapeutic targets. Our findings suggest that LMRDEGs significantly contribute to SARC progression, warranting further exploration.

We identified 17 LMRDEGs that were differentially expressed between SARC and control samples. These genes may regulate lactate metabolism and offer insights into SARC’s pathophysiology. Their involvement in related metabolic disorders and potential as therapeutic targets merit further study.

FOXO3, as a transcription factor, serves multiple biological functions^40^. It can promote the degradation of muscle proteins and atrophy by regulating pathways such as Atrogin-1/MAFbx and MuRF1^41^, while also upregulating antioxidant enzymes such as superoxide dismutase to protect against oxidative damage^42^. In this study, the upregulation of FOXO3 likely reflects the complex stress and regulatory dynamics during the progression of sarcopenia^43^, rather than exerting a singular protective or damaging effect. Regarding the upregulation of oxidative phosphorylation–related genes, although sarcopenia is typically accompanied by mitochondrial dysfunction, this study found that related genes were upregulated. This may represent a compensatory response of muscle cells to impaired energy metabolism. However, this interpretation remains speculative, as direct mechanistic evidence is lacking. Further research, including longitudinal population studies, cellular experiments, and animal models, is required to confirm these findings. Future research should focus on the dynamic regulatory roles of key genes in sarcopenia progression and their molecular mechanisms, with the aim of providing a stronger theoretical basis for diagnosis and therapeutic intervention.

This study highlights DEGs related to lactate metabolism and systematically analyzes their association with abnormal molecular pathways in sarcopenia. Enrichment analysis revealed that LMRDEGs are involved not only in energy generation and utilization pathways, such as pyruvate metabolism and oxidative phosphorylation, but also in key signaling pathways, including HIF-1 and mTOR. Lactate, as a signaling molecule, can directly upregulate HIF-1 activity, enabling muscle cells to adapt to hypoxia and metabolic stress^44^. Conversely, imbalances in lactate metabolism can disrupt energy-sensing pathways such as mTOR and AMPK, thereby altering the balance between protein synthesis and degradation^45^. Key LMRDEGs such as LDHA and LDHB are central to lactate production and utilization^46^, while PPARGC1A and FOXO3 regulate mitochondrial function and oxidative stress^47^. Abnormal expression of these molecular nodes can lead to muscle wasting and dysfunction by disrupting energy metabolism and redox balance^47,48^. In summary, lactate metabolic abnormalities may serve as a molecular link in the development of sarcopenia through the regulation of energy metabolism, oxidative stress, and signaling pathways. This study provides a theoretical framework for understanding the role of lactate metabolism in sarcopenia and identifying potential targets for intervention.

Immune infiltration analysis via ssGSEA, assessing 28 immune cell types in SARC, revealed distinct patterns of macrophage and NK cell infiltration between risk groups, consistent with immune dysregulation in muscle wasting^49^. Differential macrophage infiltration aligns with findings in age-related SARC, where polarized macrophages regulate muscle regeneration through IL-10/STAT3 signaling^50^. The observed NK cell enrichment in the LowRisk group supports their protective roles in murine models of muscle injury^51^, suggesting conserved mechanisms warranting validation. Notably, the FOXO3–plasmacytoid dendritic cell association extends FOXO3’s known immunomodulatory roles in aging, strengthening the concept of immune–metabolic crosstalk in SARC pathogenesis^52^. These immune features suggest promising therapeutic targets, particularly in macrophage polarization, an area under preclinical exploration for related myopathies^53^.

Using LASSO regression, we developed a diagnostic model incorporating five LMRGs (PPARGC1A, PIK3C2A, FOXO3, HTT, and GSR), achieving AUCs of 0.7–0.9. Inclusion of FOXO3 and PPARGC1A supports their established roles in muscle metabolism^54^, whereas the involvement of HTT aligns with its emerging role in Huntington’s disease–related myopathy^55^. The model mirrors current trends in lactate-focused diagnostics, such as in cancer cachexia^56^; however, it has some limitations. Clinical variables, such as gait speed and grip strength, which have been shown to improve diagnostic validity in the FNIH SARC Project^57^, should be integrated. Environmental modulation of LMRDEGs also deserves attention, particularly given the effectiveness of lifestyle interventions observed in the SPRINTT trial^58^.

The present study had several limitations. The small sample size may affect external validity, warranting future validation in larger cohorts. In addition, the lack of experimental validation limits mechanistic insights into the identified LMRDEGs, and potential batch effects from dataset integration should be addressed. Nevertheless, our identification of 17 LMRDEGs offers valuable insights into the molecular mechanisms of SARC and highlights potential targets for intervention.

## Conclusion

In summary, this study reveals the potential role of LMRDEGs in SARC pathogenesis. Through integrative bioinformatics analyses, we identified key genes and pathways warranting further exploration. Overall, our findings provide a foundation for future research into diagnosing SARC, developing further diagnostic models, and establishing therapeutic strategies. However, further verification through extensive independent cohorts and experimental studies remains imperative to confirm the robustness of the results.

## Supplementary Information

Below is the link to the electronic supplementary material.

Supplementary Material 1

Supplementary Material 2

Supplementary Material 3

Supplementary Material 4

Supplementary Material 5

## Data Availability

Data is provided within the manuscript or supplementary information files.
